# Treatment of Chronic Experimental Autoimmune Encephalomyelitis with Epigallocatechin-3-Gallate and Glatiramer Acetate Alters Expression of Heme-Oxygenase-1

**DOI:** 10.1371/journal.pone.0130251

**Published:** 2015-06-26

**Authors:** Antonia Janssen, Sebastian Fiebiger, Helena Bros, Laura Hertwig, Silvina Romero-Suarez, Isabell Hamann, Coralie Chanvillard, Judith Bellmann-Strobl, Friedemann Paul, Jason M. Millward, Carmen Infante-Duarte

**Affiliations:** 1 Institute for Medical Immunology, Charité—Universitätmedizin Berlin, Berlin, Germany; 2 Experimental and Clinical Research Center, joint cooperation between the Charité Medical Faculty and the Max-Delbrück Center for Molecular Medicine, Berlin, Germany; 3 NeuroCure Clinical Research Center, Charité—Universitätmedizin Berlin, Berlin, Germany; 4 Department of Neurology, Charité Universitätsmedizin Berlin, Berlin, Germany; Friedrich-Alexander University Erlangen, GERMANY

## Abstract

We previously demonstrated that epigallocatechin-3-gallate (EGCG) synergizes with the immunomodulatory agent glatiramer acetate (GA) in eliciting anti-inflammatory and neuroprotective effects in the relapsing-remitting EAE model. Thus, we hypothesized that mice with chronic EAE may also benefit from this combination therapy. We first assessed how a treatment with a single dose of GA together with daily application of EGCG may modulate EAE. Although single therapies with a suboptimal dose of GA or EGCG led to disease amelioration and reduced CNS inflammation, the combination therapy had no effects. While EGCG appeared to preserve axons and myelin, the single GA dose did not improve axonal damage or demyelination. Interestingly, the neuroprotective effect of EGCG was abolished when GA was applied in combination. To elucidate how a single dose of GA may interfere with EGCG, we focused on the anti-inflammatory, iron chelating and anti-oxidant properties of EGCG. Surprisingly, we observed that while EGCG induced a downregulation of the gene expression of heme oxygenase-1 (HO-1) in affected CNS areas, the combined therapy of GA+EGCG seems to promote an increased HO-1 expression. These data suggest that upregulation of HO-1 may contribute to diminish the neuroprotective benefits of EGCG alone in this EAE model. Altogether, our data indicate that neuroprotection by EGCG in chronic EAE may involve regulation of oxidative processes, including downmodulation of HO-1. Further investigation of the re-dox balance in chronic neuroinflammation and in particular functional studies on HO-1 are warranted to understand its role in disease progression.

## Introduction

Multiple sclerosis (MS) is a chronic inflammatory disease of the central nervous system (CNS) that represents one of the main causes of neurological disability in young adults. MS is considered an immunological disease that is initiated by CNS specific autoreactive T cells and results in demyelination and neuroaxonal damage. MS manifests in different clinical forms. The most common is the relapsing-remitting MS (RRMS) course characterized by total or partial recovery after attacks, and affecting up to 85% of the patients. However in most RRMS patients, the disease eventually converts to a secondary progressive course (SPMS) after 10–25 years of disease duration [1]. The progressive forms of MS represent about 15% of the cases and include the primary progressive (PP) and progressive-relapsing (PR) form, characterized by a continuous disease progression without relapses in PPMS, or rare superimposed relapses in PRMS.

In the progressive forms of MS, neurodegenerative processes, rather than inflammation, are presumed to be responsible for increasing clinical disability [2–4]. However, current therapeutic agents for MS exert primarily anti-inflammatory or immunomodulatory effects, and show minimal or no clinical benefit in preventing progression of neurologic disability in patients with PPMS or SPMS. There is therefore an urgent need to establish new therapies for these patients.

Evidence from MS and from the animal model, experimental autoimmune encephalomyelitis (EAE) suggests that oxidative stress and dysregulated iron metabolism contribute to neuronal damage [5–7]. We and others have shown that epigallocatechin-3-gallate (EGCG), a polyphenol derived from green tea, is capable of protecting from neuronal damage by inhibiting the formation of reactive oxygen species in neurons, as well as through iron chelating and anti-apoptotic functions [8–11]. In addition, we demonstrated in relapsing-remitting EAE that EGCG can reduce the clinical severity of EAE by both limiting brain inflammation and reducing neuronal damage [8]. More recently, Wang et al. demonstrated that EGCG exerts a protective effect on chronic EAE by altering the balance between pro- and anti-inflammatory T cell phenotypes [12]. On the other hand, it was recently shown that glatiramer acetate (GA), an immunomodulatory drug approved for RRMS, may promote neurogenesis, neuroprotection and remyelination [13–17] indicating that GA may also have therapeutic potential in treating the progressive forms of MS. In this context, we demonstrated in the mouse model of RRMS that the therapeutic effects of EGCG synergize with the effects of GA [18]. Importantly, we showed that GA not only enhanced the anti-inflammatory effects of EGCG, but also its neuroregenerative potential.

Therefore, in light of the neuroprotective properties of both compounds, we hypothesized that the combination therapy of EGCG and GA would have a stronger effect than EGCG alone, not only modulating inflammation, but also synergistically promoting neuroprotection and neuroregeneration in chronic EAE. To test this hypothesis, we applied single and combination therapies of GA+EGCG to a model of chronic progressive EAE. We show that while GA or EGCG alone improved EAE, the combined treatment has no effect on EAE progression.

## Materials and Methods

### Animals. Induction and treatment of EAE

Mice were acquired and cared for in accordance with the guidelines published in the *NIH Guide for the Care and Use of Laboratory Animals* (NIH Publication No. 85–23, revised 1985), and the principles presented in the *Guidelines for the Use of Animals in Neuroscience Research* by the Society for Neuroscience (published in Membership Directory of the Society, pp. xxvii-xxviii,1992). All experiments were conducted in accordance with the ARRIVE guidelines, and all experimental procedures were approved by the regional animal study committee of Berlin, the *Landesamt für Gesundheit und Soziales Berlin* (LAGeSo).

C57BL/6 mice were bred and maintained in the facilities of the “Forschungsinstitut für Experimentelle Medizin” (FEM, Charité—Universitätsmedizin, Berlin, Germany), under specific pathogen-free (SPF) conditions. EAE was induced as described previously [19]. In brief, 8–10 week old female C57BL/6 mice were immunized subcutaneously with 250 μg of MOG_35–55_ (Pepceuticals, Leicester, UK) emulsified in an equal volume of PBS and complete Freund’s adjuvant (Difco, Franklin Lakes, NJ) containing 800 μg Mycobacterium tuberculosis H37Ra (Difco). 200 ng of Bordetella pertussis toxin (List Biological Laboratories, Campell, CA) was administered intraperitoneally at day 0 and 2. EAE experiments were carried out in our animal facilities under standard husbandry conditions.

Mice were weighed and scored daily as follows: 0 = no disease; 1 = complete tail paralysis; 2 = hindlimb paresis; 3 = hindlimb plegia; 4 = paraplegia and forelimb weakness; 5 = moribund or death due to EAE. Mice were euthanized when they reached a score > 3 (hindlimb plegia) or when they lost more than 20% of the initial body weight. At time of termination of the experiments, mice were sacrificed under deep anesthesia. None of the mice reached moribundity during the studies.

To investigate the therapeutic potential of the combination therapy with GA and EGCG, mice were randomized in 4 treatment groups (control, EGCG, GA, EGCG+GA), when each individual animal reached a clinical score ≥ 1 as described previously [18]. EGCG (Sigma-Aldrich, Deisenhofen, Germany) was dissolved in 0.9% NaCl. 150 μg GA (TEVA) or vehicle (mannitol 4%) was dissolved in PBS, emulsified in incomplete Freund’s adjuvant (Difco) and injected s.c. once.

The control group (n = 17) received a single injection of 4% mannitol (vehicle control for GA) and oral applications of 0.9% NaCl twice daily (vehicle control for EGCG). The EGCG (n = 18) group received a single s.c. mannitol injection at treatment initiation and was further treated with 300 μg EGCG given by oral gavage twice daily for a period of 50 days i.e. until day 62 after immunization. In the GA group (n = 17), a suboptimal GA dose (150 μg, TEVA) was dissolved in PBS, emulsified in incomplete Freund’s adjuvant (Difco) and injected s.c. at treatment initiation. This group was treated twice daily with 0.9% NaCl for the complete experimental period. The EGCG+GA (n = 17) received one single injection of GA; EGCG was administered by oral gavage twice per day for a period of 50 days. Sample size estimates were based on our previous studies with the same drugs [8,18], and with the support of the Biostatistics Department of the Charité.

To elucidate potential mechanisms of action and interference of GA and EGCG, treatment was started at day 14 post-immunization in animals showing the first clinical signs (tail paresis). EGCG (+ one injection of 4% mannitol) or EGCG+GA treated mice (n = 26, 13 mice per group) were treated for a defined period of exactly 12 days and sacrificed afterwards. At that time point, earlier clinical effects of the treatments were already manifested.

To confirm findings on HO-1 expression in EGCG versus EGCG+GA treated groups, an additional EAE experiment was performed including a vehicle-treated control group treated with one injection of 4% mannitol and two daily application of 0.9% NaCl (n = 33, 11 mice per group). Treatment started when each individual animal first showed an EAE score ≥1 (the first at day 14 p.i.) and was applied for 12 days.

Apart from monitoring the treatment effect on the clinical outcome, the effects of the drugs and drug combination on iron homeostasis, neurodegeneration and inflammation (at tissue, cellular and molecular levels) were assessed.

### Histology

At the time of sacrifice, mice were transcardially perfused with PBS, and tissues removed for fixation in zinc fixation solution, as described previously [20,21]. The tissues were cryoprotected overnight in 30% sucrose. Spinal cords were cut into 8 cross-sectional segments embedded in Tissue Tek (Sakura), frozen in a methylbutane/dry ice mixture, and then cut into 12 μm sections with a cryostat. Sections were stained with hematoxylin and eosin (H&E), Luxol Fast Blue (LFB) and Bielschowsky silver staining, according to standard methods, and sections were examined by light microscopy for the presence of inflammation, demyelination or axonal damage, respectively. Semi-quantitative assessment was done by scoring quadrants of each of the eight transverse segments for inflammatory infiltrates (H&E; 0 = no inflammation; 1 = mild inflammation; 2 = severe inflammation), demyelination (LFB; 0 = no demyelination; 1 = mild demyelination, 2 = severe demyelination) or axonal damage (Bielschowsky; 0 = no axonal damage; 1 = mild axonal damage; 2 = severe axonal damage). Sections for each mouse were assessed in a blinded manner by two independent observers. Data are presented as the percentage of total quadrants positive for inflammatory infiltrates, demyelination or axonal damage relative to all assessed quadrants. Pictures were taken with a Zeiss Observer Z1, AxioCamICc 1.

### Proliferation Assay

Proliferation of MOG-specific CD4^+^ T-cells was assessed using the carboxyfluorescein succinimidyl ester (CFSE) dilution assay. Draining axillary and inguinal LNs were removed and homogenized at day 62 or day 26 post-immunization. LN cells (1x10^6^/ml) were washed and resuspended in pre-warmed RPMI+1% Hepes (RH) and incubated with 2.5 μM CFSE (Molecular Probes, Germany) at 37°C for 10 min. The staining was quenched with addition of culture media containing 10% FCS. Cells were placed in a 96 round bottom plate (2×10^5^ cells/well) and cultured for 72 h in the presence of 50 μg/ml MOG_35–55_ (purity >95%, Pepceuticals, Leicester, UK). Cells cultured alone, in the absence of antigen served as the negative control. As a positive control, cells labeled with CFSE were cultured in 96-well plates coated with 3 μg/ml anti-CD3 and 2.5 μg/ml anti-CD28 antibodies (BD Biosciences, Heidelberg, Germany) for 72 h. Cell division was analyzed using flow cytometry (FACS Canto, BD), gating on T cells, based on staining with anti-CD4 Alexa Fluor 647 (Invitrogen, Darmstadt, Germany).

### Intracellular cytokines and FoxP3 staining

To assay the function of CD4^+^ T cells, mice were treated for 12 days with vehicle, GA, EGCG or the combination therapy. At day 26 after immunization, mononuclear cells from the spleen (2×10^6^/ml) and the CNS were cultured and stimulated with 3 μg/ml anti-CD3 and 2.5 μg/ml anti-CD28 (BD Biosciences) for 4 h at 37°C. Brefeldin A at 1 μ/ml was added for the last 2 h of incubation. After incubation cells were blocked with antibodies to the FcγIII/II receptors (anti-mouse CD16/CD32, BD Bisosciences) to avoid nonspecific staining and were subsequently stained with PerCP-labeled anti-CD4 (BD Biosciences) for spleen and against CD4 and CD45 (PE Cy7, BD Biosciences) for CNS according to standard procedures followed by fixation using Fix/Perm Buffer (eBioscience, Frankfurt, Germany) for 24 h. To determine T cell phenotype and the magnitude of cytokine production, T cells were then stained with anti-IL17 PE (BD Biosciences), anti-IFN gamma eFluor 450 (eBioscience) and anti-FoxP3 APC (eBioscience). FACS analysis was performed on a FACS Canto.

### Quantitative Real-time RT PCR

Brains were separated into anterior and posterior regions by a coronal cut at the junction of the cerebrum and cerebellum, and another cut 4mm anterior to this, with the guidance of a mouse brain mould. Total RNA was extracted from zinc-fixed brain tissue using the guanidinium thiocyanate method, with the peqGOLD TriFast reagent (Peqlab, Erlangen, Germany), according to the manufacturer’s instructions. The RNA was reverse transcribed, and quantitative PCR (qPCR) carried out using an ABI Prism 7000 Sequence Detection System (Applied Biosystems). Primers and probes (Table 1) were from Eurofins MWG Operon (Ebersberg, Germany). Cycle threshold values were converted to arbitrary units using a standard curve and data are reported as the ratio of target gene expression over 18s rRNA, which served as the endogenous reference. In some cases, samples were excluded from the analysis due to poor quality of the RNA.

**Table 1:** Primers and probes for qPCR.

18s	forward	TTC GAA CGT CTG CCC TAT CAA
	reverse	TCC CCG TCA CCC ATG GT
	probe	CGA TGG TAG TCG CCG TGC CTA CCA
HO-1	forward	GTG ATG GAG CGT CCA CAG C
	reverse	TGG TGG CCT CCT TCA AGG
	probe	CGA CAG CAT GCC CCA GGA TTT GTC
T-bet	forward	CAA CAA CCC CTT TGC CAA AG
	reverse	TCC CCC AAG CAG TTG ACA GT
	probe	CCG GGA GAA CTT TGA GTC CAT GTA CGC
RORγT	forward	CCG CTGA GAG GGC TTC AC
	reverse	TGC AGG AGT AGG CCA CAT TAC A
	probe	AAG GGC TTC TTC CGC CGC AGC CAG CAG
IFNγ	forward	CAG CAA CAG CAA GGC GAA A
	reverse	CTG GAC CTG TGG GTT GTT GAC
	probe	AGG ATG CAT TCA TGA GTA TTG CCA AGT TTG A
IL-17	forward	TCA TCT GTG TCT CTG ATG CTG TTG
	reverse	TCG CTG CTG CCT TCA CTG T
	probe	TGC TGC TGA GCC TGG CGG C

### Serum iron assay

Soluble iron content in blood serum was quantified by using a modification of the ferrozine-based assay previously described [22]. Briefly, 50 μL of serum samples were mixed with equal volumes of HCl 10 mM, then incubated for 30 min with 20 μL of an iron detection reagent (6.5 mM ferrozine, 6.5 mM neocuproine, 2.5 M ammonium acetate and 1 M ascorbic acid, all purchased from Sigma-Aldrich, dissolved in water). Absorbance was measured at 560 nm on a microplate reader (GloMax-multi microplate multimode reader, Promega). The amount of iron was calculated by comparing the absorbance of the samples with that of a FeCl_3_ standard curve.

### Statistics

EAE disease courses, histology scores, and cumulative disease activity were analyzed with the non-parametric Kruskal-Wallis ANOVA with the Dunn’s post-hoc test, or the Mann-Whitney U test. Other data was analyzed using the t-test or ANOVA, as appropriate. p-values <0.05 were considered significant. GraphPad Prism version 5.01 (GraphPad Software, San DiegoCA) was used for the analysis. *p<0.05, **p<0.01, ***p<0.001.

## Results

### EGCG and GA single therapy ameliorates chronic EAE, but the combination therapy does not

EGCG has been shown to have both a protective and therapeutic effect on relapsing remitting EAE [18], as well as to be protective in MOG-induced chronic EAE [23]. Here, we evaluated the therapeutic potential of EGCG alone in already established MOG-induced chronic EAE and tested whether the combination therapy of EGCGC and GA could be beneficial in this EAE model. GA is an approved MS therapy, with well-established efficacy in ameliorating EAE. Therefore, in the current experimental design we employed a suboptimal dose of GA, in order that any potential synergistic effects with EGCG would not be obscured by the efficacy of GA. Mice were randomly assigned into the 4 treatment groups after immunization when animals developed a clinical score ≥ 1 (tail paralysis). Treatment groups included a control group (animals treated with the vehicles, NaCl and mannitol), an EGCG treated group (that also received a single injection of mannitol), a GA treated group (that also received daily applications of 0.9% NaCl) and an EGCG+GA treated group. EGCG (300 μg) or vehicle (0.9% NaCl) were given by oral gavage twice daily for a period of 50 days. A suboptimal GA dose (150 μg, TEVA) or vehicle (mannitol 4%) was dissolved in PBS, emulsified in incomplete Freund’s adjuvant (Difco) and injected s.c. The day of treatment initiation did not significantly differ between the groups (Table 2).

**Table 2:** Treatment onset and mean maximum clinical score.

Group	Day of treatment start (mean±sem)	*P*-value (ANOVA)	Maximum score (mean±sem)	*P*-value (Kruskal-Wallis)
Mannitol+NaCl	11.94 ± 0.4242		3.00 ± 0.20	
GA+NaCl	12.67 ± 0.5542		2.50 ± 0.17	
Mannitol+EGCG	12.72 ± 0.4339		2.42 ± 0.18	
GA+EGCG	12.00 ± 0.3792		3.05 ± 0.90	
		**0.4702**		**0.0272**

**Fig 1:**
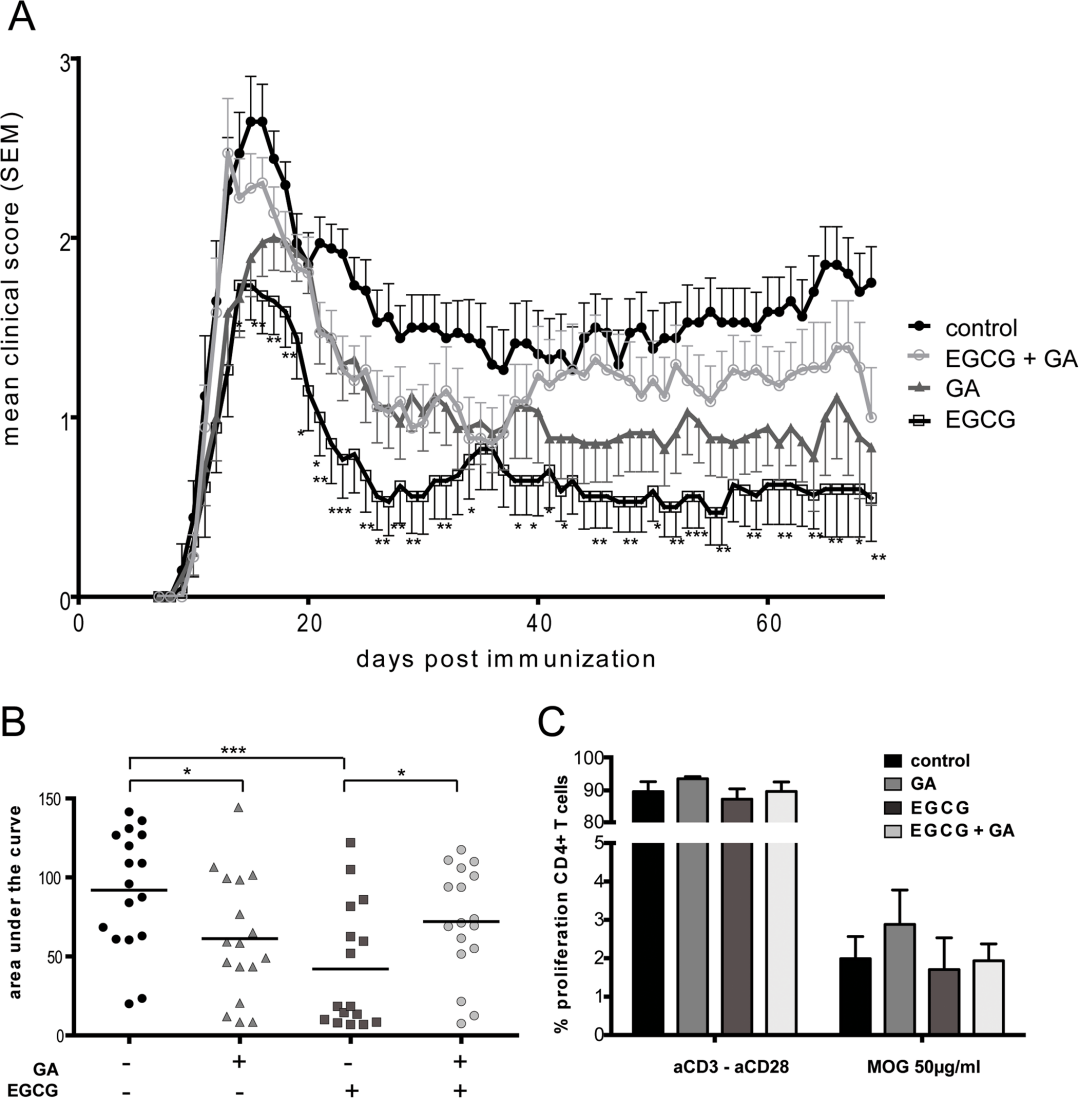
Effects of EGCG alone and in combination with a suboptimal dose of GA in established EAE. A: Disease severity of control, GA, EGCG and combination therapy group. Analysis includes data from three independent experiments (Mann-Whitney test). B: Mean clinical scores of animals are shown. Data are given as mean ± SEM. Cumulative disease activity is represented as the area under the curve of control, GA, EGCG and combination therapy group (Kruskal-Wallis). C: Proliferation of MOG-specific CD4^+^ T cells at day 62 after immunization. CFSE-labeled lymph node cells were incubated for 72h with MOG (50μg/ml). As a positive control, cells were cultured with 3 μg/ml anti-CD3 antibody and 2.5 μg/ml anti-CD28 antibody. For the negative control, cells were incubated alone, in the absence of antigen. To assess cell division cells were stained with anti-CD4 Alexa Fluor 647 and analyzed by flow cytometry (ANOVA). *p<0.05, **p<0.01, ***p<0.001.

Vehicle-treated control mice developed the expected chronic EAE course, reaching a disability plateau at about day 20 post- immunization. Mice treated with EGCG alone showed a significant reduction on chronic EAE severity (Fig 1A). This effect was sustained over the entire period of observation, as indicated by the reduced mean clinical score (Fig 1A). Additionally, even a single suboptimal application of GA alone showed a therapeutic effect. As expected with this suboptimal treatment regime, this effect was only transient, and GA-treated mice reached a score similar to the control animals around day 10 after GA injection, in contrast to the sustained benefit with daily EGCG treatment. However, contrary to our expectations, the combination therapy of GA and EGCG did not improve the EAE clinical course when applied to animals with established disease. Rather, it appears that GA interfered with EGCG, abolishing its beneficial effects on established EAE (Fig 1A). The therapeutic effects of the single and the combination therapies on EAE severity are more clearly seen on the plots comparing the cumulative disease activity of the different treatment groups over the course of the entire experiment (Fig 1B). There was also a significant difference between treatment groups in the mean maximal EAE score (Table 2).

To investigate whether the interference of EGCG effects by GA may have occurred at the level of encephalitogenic T cell responses, we monitored the *ex vivo* response to MOG of T cells extracted from the lymph nodes of the mice from the experiment depicted in Fig 1A and 1B. The data indicate that the response to MOG did not significantly differ between mice treated with EGCG alone and those treated with the combination of GA and EGCG. Thus, the interference of GA on EGCG effects seems not to affect the proliferative capacities of the encephalitogenic T cells, at least at this late time point (Fig 1C). Similarly, we did not observe treatment effects on MOG-induced T cell proliferation at an earlier time point, day 16 post-immunization, i.e. after 10 days of treatment (data not shown).

### A single application of GA in combination with EGCG alters the neuroprotective effects of EGCG in chronic EAE

**Fig 2:**
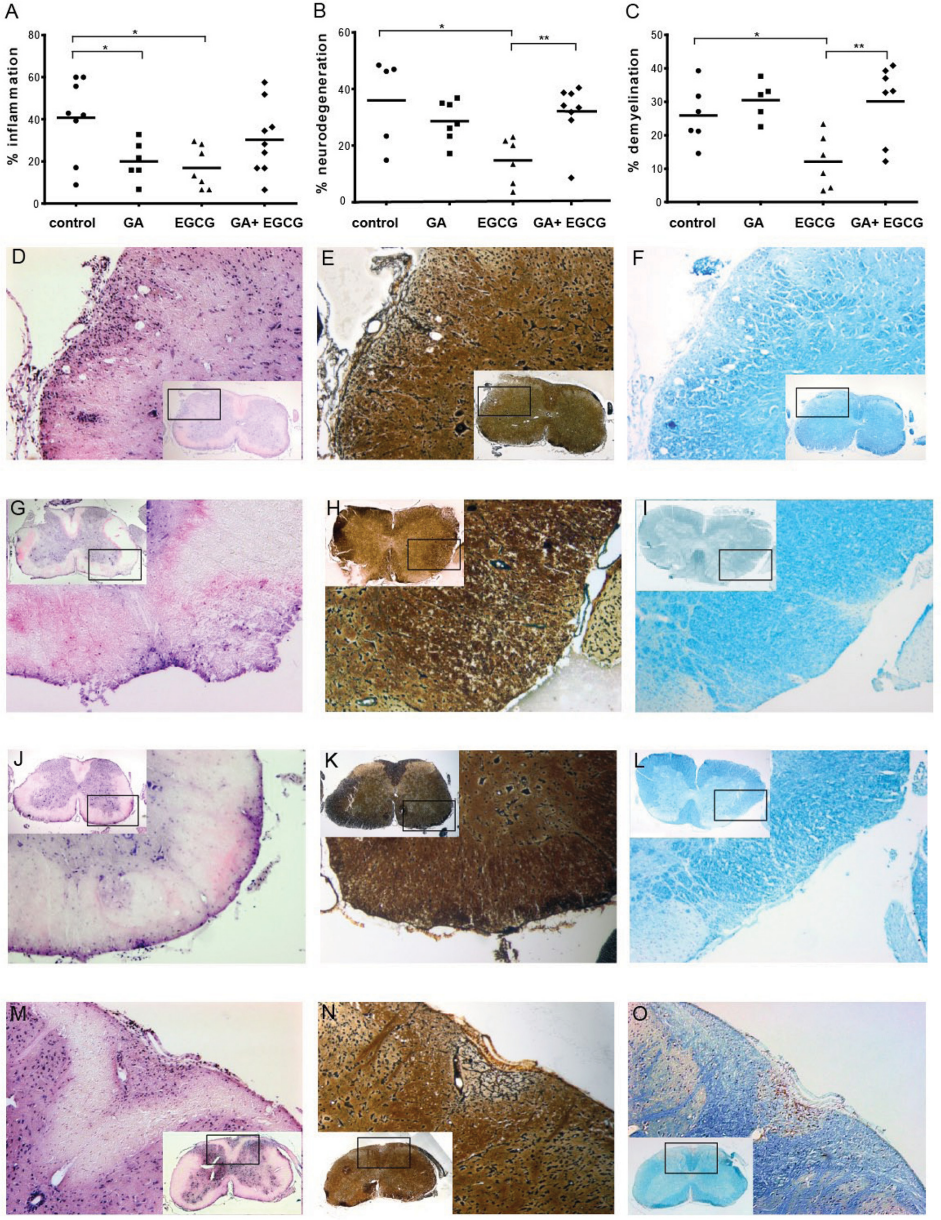
The application of GA in combination with EGCG interferes with the anti-inflammatory and neuroprotective effects of EGCG single therapy. A: Quantification of inflammation, B: neurodegeneration C: and degree of demyelination in the spinal cord from control, GA, EGCG and combination therapy group included in Fig 1. Histopathological changes were assessed semi-quantitatively as percentage of spinal cord quadrants that showed pathological changes related to all investigated tissue quadrants. Representative Hematoxylin and Eosin staining to monitor inflammation of transverse spinal cord sections of control (D), GA treated mice (G), EGCG treated mice (J) and mice treated with the combination therapy (M) are shown. Bielschowsky staining was used to assess axonal damage. Representative spinal cord sections of control (E), GA treated mice (H), EGCG treated mice (K) and mice treated with the combination therapy (N) are shown. The degree of demyelination was determined by Luxol Fast Blue staining. Representative LFB spinal cord sections of control group (F), GA treated group (I), EGCG treated group (L) and group treated with EGCG and GA (O) are shown. (Kruskal-Wallis) *p<0.05, **p<0.01.

To further examine potential mechanisms of drug interference in the chronic EAE model, we performed histopathological analysis of spinal cord sections from the mice included in the experiment shown in Fig 1. We focused attention on the effects of the different therapy regimes on inflammation, demyelination and neurodegeneration. Staining with hematoxylin and eosin revealed that both GA and EGCG single treatments led to significant reductions in spinal cord inflammation, compared to the vehicle control group (Fig 2A). However, mice treated with the combination therapy showed no significant reduction in inflammation compared to controls (Fig 2A). Interestingly, EGCG single treatment also preserved axons and myelin during chronic EAE, as reflected by the staining of neurofilaments assessed by Bielschowsky silver staining (Fig 2B), and myelin determined by LFB staining (Fig 2C). The single suboptimal application of GA was not sufficient to preserve axons and myelin. Moreover, the EGCG-mediated protection from both axonal damage and demyelination was abolished by the single application of GA (Fig 2B and 2C).

### GA does not influence the anti-inflammatory effects of EGCG treatment in vivo

**Fig 3:**
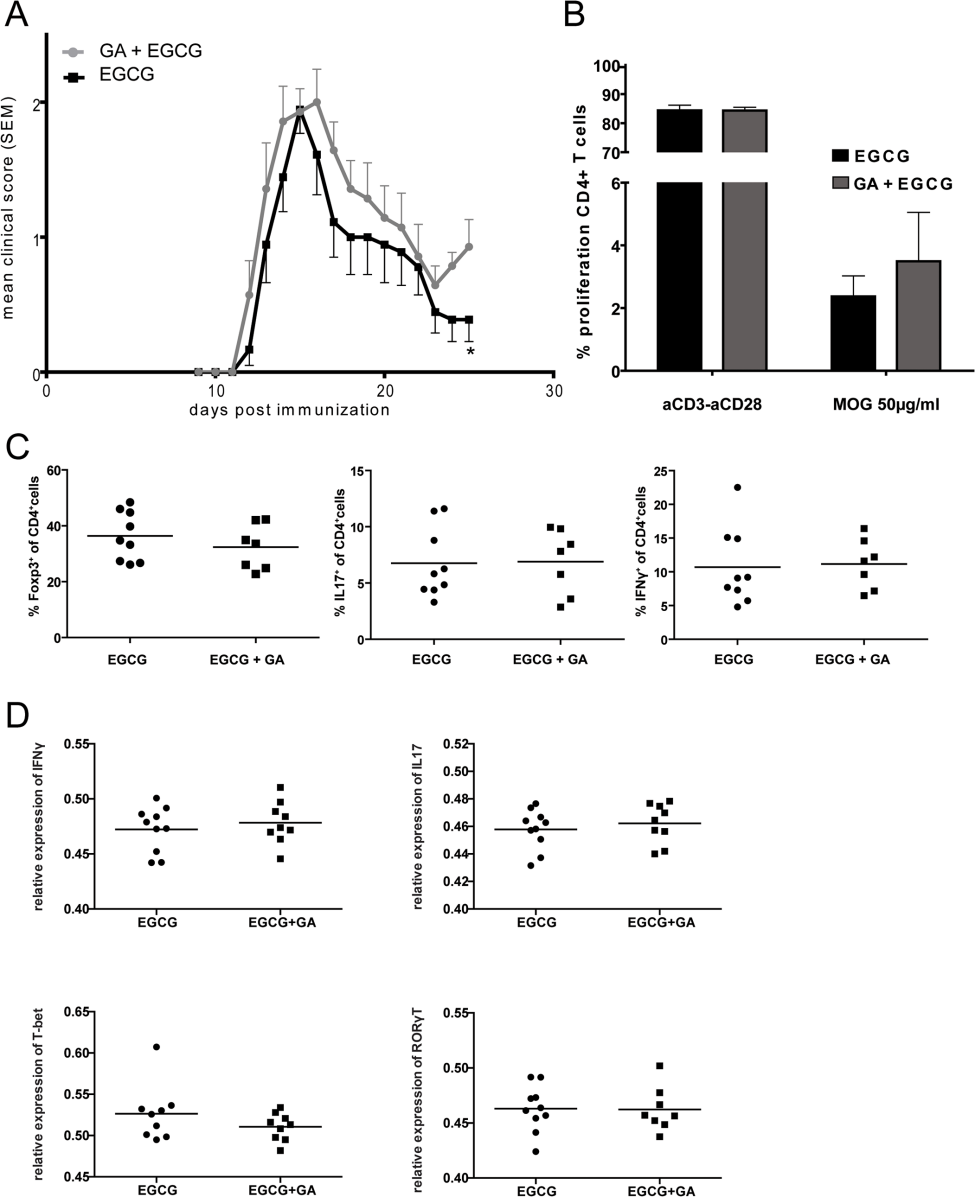
GA does not influence the anti-inflammatory effects of EGCG-treatment *in vivo*. To elucidate mechanisms of action of EGCG, mice with a minimum score of 0.5 were treated for 12 days with EGCG alone or with combination of EGCG and GA, starting at day 14 after immunization. A: Disease severity of EGCG and combination therapy group. Analysis includes data from two independent experiments (Mann-Whitney). B: Proliferation of MOG-specific CD4^+^ T-cells day 26 after immunization. C: Mononuclear cells from CNS were stimulated with anti-CD3 and anti-CD28. Cells were stained with anti-CD4, anti-CD45 as surface markers and with anti-IFNγ, anti-FoxP3 and anti-IL17. d) RNA extracted from the CNS tissue (brains and spinal cords) was used to determine T-bet, RORγt, IFNγ and IL17 mRNA expression using quantitative real-time RT-PCR. (t-test) *p<0.05.

To elucidate the mechanisms by which GA may interfere with EGCG, we performed additional EAE experiments to thoroughly compare the effects of treatment with EGCG and EGCG+GA. In order to facilitate the identification of potential early mechanisms of drug interference, affected mice displaying the first signs of disability (tail paresis, EAE score = 1) at day 14 post-immunization were treated with EGCG alone or with EGCG+GA until clinical differences between the two treatment groups became significant (treatment period of 12 days) (Fig 3A).

We then assessed the proliferative capacities of encephalitogenic T cells from the treated animals *ex vivo* using the CFSE dilution assay. As shown in Fig 3B, peripheral CD4+ T cells of GA+EGCG-treated mice showed a slight but not significantly increased proliferation to MOG compared with EGCG-treated animals.

It has been reported that EGCG modulates T cell differentiation in MOG-induced EAE and *in vitro*. Thus, we monitored the frequencies of regulatory T cells as well as IL-17 and IFN-gamma producing T cells in the CNS of EGCG and GA+EGCG-treated animals. As shown in Fig 3C, no differences were found between the treatment groups in terms of the phenotype of infiltrating CD4-positive T cells. Since both IFN-gamma and IL-17 may be expressed by infiltrating lymphocytes other than CD4+T cells, we also analyzed gene expression of these key inflammatory cytokines and their corresponding transcription factors T-bet and RORgammaT using mRNA extracted from the CNS tissue of the treated animals. No significant differences were detected in the expression of the inflammatory mediators IFN-gamma and IL-17, or in the expression of the corresponding transcription factors T-bet or RORgammaT (Fig 3D). This analysis corroborated the data obtained at the single cell level (Fig 3C). Thus, in terms of (anti)-inflammatory parameters, no differences were detected between the two treatment groups.

### The combined application of GA and EGCG treatment does not alter the iron chelator activity of EGCG

**Fig 4:**
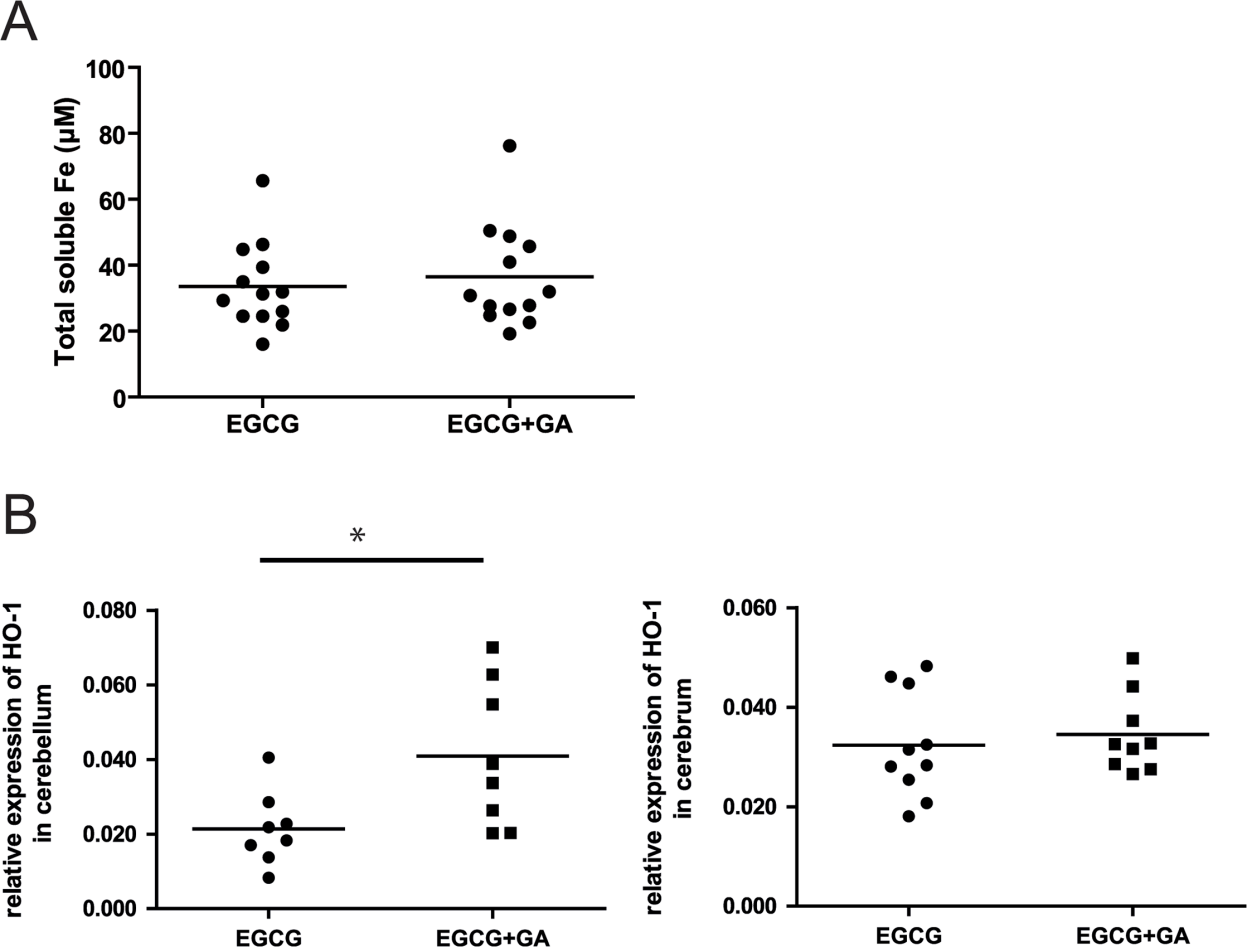
The combined application of GA and EGCG treatment does not alter the iron chelator activity but enhanced HO-1 expression compared to EGCG. A: Soluble iron content in blood serum was quantified day 26 after immunization by using a modification of the ferrozine-based assay. B: Relative mRNA expression of HO-1 in cerebellar and cerebral regions of the CNS of mice included in Fig 3 treated with EGCG alone, or in combination with GA. (t-test) *p<0.05.

Evidence from EAE and MS suggests that oxidative stress and dysregulated iron metabolism contribute to neuronal damage. EGCG has the potential to impact these two processes by minimizing the amount of iron ions available to generate destructive oxygen radicals [10]. Therefore, to explore whether GA may interfere with the iron chelating activity of EGCG, soluble iron content in blood serum of the different treatment groups was quantified using a ferrozine-based assay. As shown in Fig 4A, there was no difference of total soluble iron detectable between the ECGC-treated and the EGCG+GA-treated groups.

### EGCG+GA alters HO-1 regulation by EGCG alone in chronic EAE

HO-1 expression has been shown to be increased in EAE and MS [24] and can be regulated by EGCG *in vitro*. We investigated HO-1 gene expression in the cerebellum and brainstem of the mice included in Fig 3 that were treated with EGCG or with EGCG+GA using real-time RT-PCR. Contrary to our expectations, addition of GA to EGCG increased HO-1 expression in the cerebellum and brainstem region compared to mice treated with EGCG alone (Fig 4B). However, in the cerebral (anterior) region of the brain, which is known to be less affected by EAE in this model, the expression of HO-1 did not differ between treatment groups. These results were unexpected, considering that EGCG is assumed to exert its antioxidant function in part by promoting HO-1 expression.

### EGCG modulates HO-1 gene expression in spinal cord and cerebellum of EAE mice

**Fig 5:**
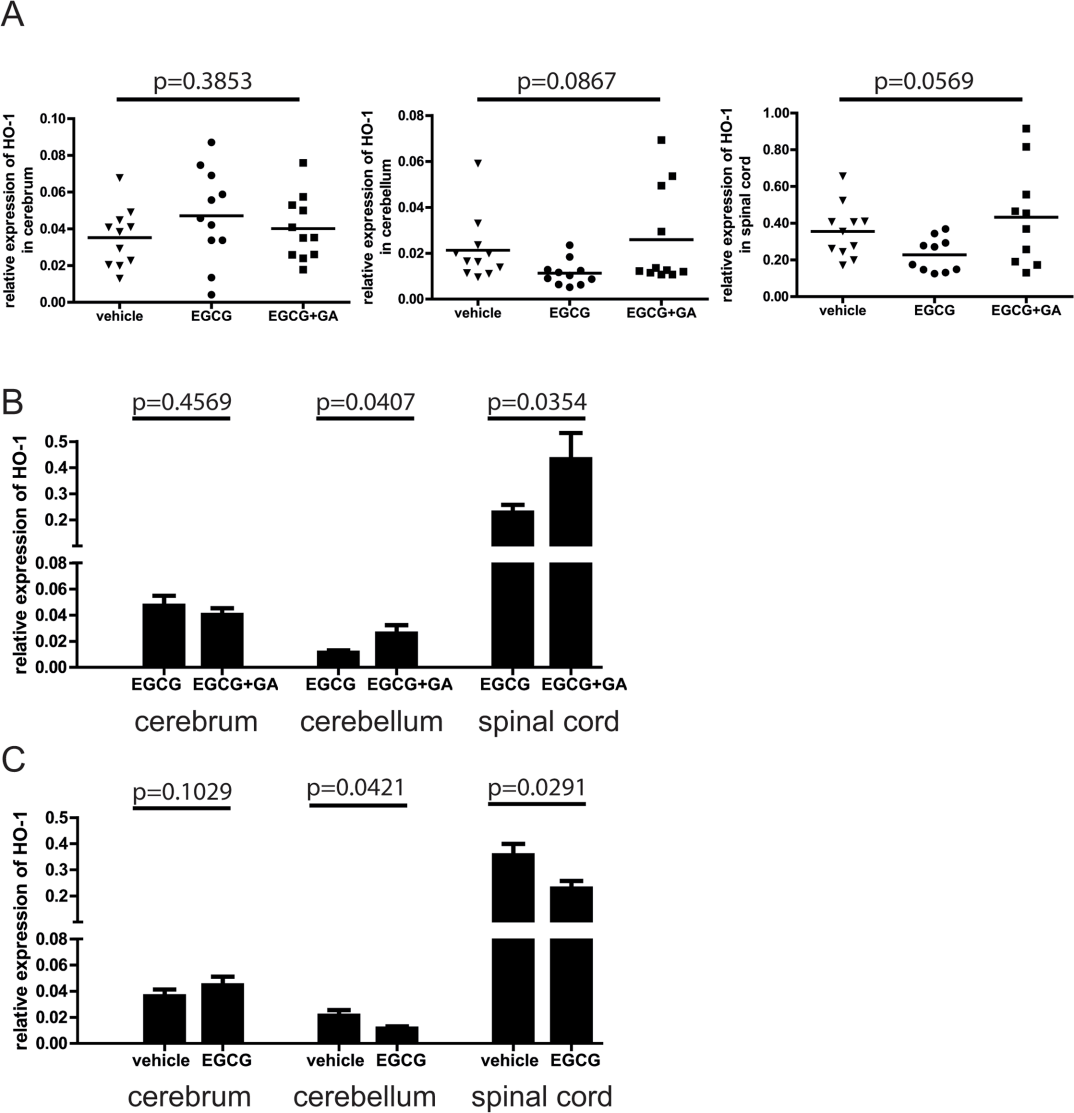
Addition EGCG therapy leads to decreased expression of heme oxygenase-1 (HO-1). A: Relative mRNA expression of HO-1 in cerebral and cerebellar regions as well as spinal cords of mice treated with vehicle control, EGCG alone, or in combination with GA. (ANOVA). B: Relative expression of HO-1 in mice treated with EGCG compared to mice treated with EGCG+GA C: Relative expression of HO-1 in mice treated with EGCG compared to control vehicle treated animals (t-test).

Thus far, we observed alteration of HO-1 expression in EAE mice treated with EGCG alone compared to EAE mice treated with EGCG+GA. To elucidate how EGCG regulates HO-1 compared to control EAE mice *in vivo*, we conducted an additional experiment with 3 treatment groups, vehicle treated, EGCG treated and EGCG+GA-treated animals. As in the experiments shown above, treatment started when mice developed a clinical score ≥ 1. Mice were treated for a period of 12 days. Here, we examined HO-1 expression in cerebrum, cerebellum and spinal cord (Fig 5A). Multiple group comparisons indicated that treatment with EGCG+GA appeared to increase expression of HO-1 in cerebellum and spinal cord relative to mice treated with EGCG alone, although this was at the edge of statistical significance. Consistent with our previous results, there was no difference in HO-1 expression in the cerebrum. Upon analyzing the data with a two-group comparison, we could confirm that treatment with EGCG+GA increased HO-1 expression in cerebellum and spinal cord when compared with EGCG alone (Fig 5B). Furthermore, these data showed that EGCG reduced HO-1 expression in the cerebellum and spinal cord as compared with the vehicle treated control group (Fig 5C).

## Discussion

We investigated whether the application of GA with EGCG would improve the therapeutic efficacy of EGCG alone in chronic neuroinflammation. Unexpectedly, we observed that the combined therapy failed to reduce the severity of chronic EAE, despite the fact that each single therapy was effective. Surprisingly, even a single administration of a supoptimal dose of GA had a sustained effect, interfering with the positive disease-limiting actions of EGCG. Interestingly, the addition of GA to EGCG appears to alter the EGCG-related downregulation of the stress-inducible enzyme HO-1.

We and others have previously reported that the prophylactic administration of EGCG ameliorated the course of both relapsing remitting EAE and chronic EAE primarily by altering the inflammatory response to PLP and MOG, respectively, and by inhibiting ROS formation [8,12]. These reports show evidence for a therapeutic effect of EGCG on already established EAE, although it remains unclear whether the therapeutic benefits relied on the same immunomodulatory mechanisms as in the preventative paradigms. Here, we demonstrated by histology that in established disease in the chronic EAE model, the therapeutic benefits of EGCG (Fig 1) were associated with prevention of demyelination and axonal injury, while the single GA dose exerted principally anti-inflammatory effects.

In contrast to our expectations, the combination treatment with EGCG with a single dose of GA had no effect on the EAE activity, probably because GA counteracts the neuroprotective effects seen under EGCG-treatment alone. However, this result cannot be explained by a disease aggravating effect of GA in chronic EAE. In our hands, a single subcutaneous injection of GA was sufficient to induce a transient improvement of the EAE course and a mild yet significant amelioration of the cumulative disease activity. This corresponded with a decrease in leukocyte infiltration in the CNS of the GA-treated animals. Although we injected only one dose of 150 μg GA, these results are in agreement with the previously reported anti-inflammatory properties of GA in mouse and rat EAE [25,26]. It is also possible that EGCG might interfere with the actions of GA. Given the oral administration of EGCG, systemic effects might interfere with the immune recognition of GA which could lead to a neutralization of its therapeutic effects.

In view of this unexpected neutralization of the positive effects of EGCG when combined with GA treatment, we further examined this treatment regime to elucidate potential molecular mechanisms behind this observation.

To explore the pathways of this interference at an earlier phase of disease, we conducted two additional EAE experiments in which mice were randomly treated with either EGCG alone or GA+EGCG, until significant differences in EAE clinical scores were observed. These EAE mice showed a generally milder disease course, than the typical course of chronic EAE. Nevertheless, only those mice with clinical signs were included in the treatment groups, therefore this experiment was a suitable comparison of these two treatments. Using this setup, we showed that GA did not influence the effects of EGCG on the expression of inflammatory mediators, and did not interfere with the iron chelating activity of EGCG. However, we showed that the application of EGCG+GA induced an increase in HO-1 gene expression in the cerebellum (but not in the cerebrum), compared to mice treated with EGCG alone. These results were confirmed in an additional treatment experiment including a vehicle control group. Here, we also examined the spinal cord of the treated EAE animals and demonstrated that HO-1 expression was particularly elevated in the spinal cords of animals treated with the combination of EGCG+GA. Moreover, contrary to expectations, treatment of EAE mice with EGCG alone decreased the expression of HO-1 in cerebellum and spinal cord when compared to the vehicle control group. In EAE, HO-1 levels correlate with clinical disability, and the enzyme is primarily expressed by inflammatory cells in CNS lesions [27–29]. Thus, one may speculate that levels of HO-1 are independent of the treatment, and simply reflect the degree of inflammation and clinical severity. Nevertheless, in our model GA did not affect the extent of CNS immune cells infiltration in treated mice, as shown in Fig 3, suggesting that HO-1 modulation under different treatments is not merely a reflection of the magnitude of inflammation.

The role of HO-1 in EAE and in neurodegenerative disorders remains controversial. While in Lewis rats inhibition of HO-1 prior to EAE onset aggravated the disease course [29], the inhibition of HO-1 in established mouse EAE had a beneficial effect [30]. HO-1 deficient mice developed a more severe EAE course when compared with wildtype animals, and induction of HO-1 after EAE onset by cobalt protoporphyrin IX showed a therapeutic effect in both relapsing-remitting and chronic EAE [30]. However, sustained induction of HO-1 by cobalt protoporphyrin IX [31], and overexpression of HO-1 in general [32] has been shown to lead to pathologic iron deposition and mitochondrial autophagy, processes that have been associated with the pathology of neurodegenerative diseases [33] and also with MS [34] and EAE [5]. Thus, HO-1 induction may be beneficial or detrimental depending on the disease stage. As postulated by Stahnke et al. stress-induced HO-1 may initially play a protective role, while its chronic upregulation, might contribute to oligodendroglial cell death and to disease progression [35]. In addition to the diverse mechanisms operating in different diseases and their animal models, the contributions of genetic differences between mouse background strains should not be underestimated, as outlined by the divergent responses to EGCG+GA combination therapy in B6 mice in the present study compared with our previous studies in SJL mice.

In summary, our results highlight the complexity of HO-1 regulation and iron homeostasis in chronic neuroinflammatory processes. Further studies on the implications of HO-1, especially in progressive EAE are clearly necessary. This may help in the designing of novel treatment strategies for the progressive forms of MS.
